# Thyroid hormone: sex-dependent role in nervous system regulation and disease

**DOI:** 10.1186/s13293-021-00367-2

**Published:** 2021-03-08

**Authors:** Shounak Baksi, Ajay Pradhan

**Affiliations:** 1 Causality Biomodels, Kerala Technology Innovation Zone, Cochin, 683503 India; 2 Biology, The Life Science Center, School of Science and Technology, Örebro University, SE-701 82 Örebro, Sweden

**Keywords:** Nervous system, Sex-specific, Dimorphic, Brain, Hypothyroidism

## Abstract

Thyroid hormone (TH) regulates many functions including metabolism, cell differentiation, and nervous system development. Alteration of thyroid hormone level in the body can lead to nervous system-related problems linked to cognition, visual attention, visual processing, motor skills, language, and memory skills. TH has also been associated with neuropsychiatric disorders including schizophrenia, bipolar disorder, anxiety, and depression. Males and females display sex-specific differences in neuronal signaling. Steroid hormones including testosterone and estrogen are considered to be the prime regulators for programing the neuronal signaling in a male- and female-specific manner. However, other than steroid hormones, TH could also be one of the key signaling molecules to regulate different brain signaling in a male- and female-specific manner. Thyroid-related diseases and neurological diseases show sex-specific incidence; however, the molecular mechanisms behind this are not clear. Hence, it will be very beneficial to understand how TH acts in male and female brains and what are the critical genes and signaling networks. In this review, we have highlighted the role of TH in nervous system regulation and disease outcome and given special emphasis on its sex-specific role in male and female brains. A network model is also presented that provides critical information on TH-regulated genes, signaling, and disease.

## Introduction

The thyroid gland is one of the earliest endocrine organs that can be observed at twenty paired somites stage in a developing human embryo [1]. Thyroid hormones (THs) are first detected in the human fetal circulation at 11–13 gestation weeks [2]. The thyroid is the only endocrine gland that can produce and store thyroid hormones (THs), triiodothyronine (T3) and thyroxine (T4). T4 is the major TH secreted by the thyroid gland, whereas T3 is the main biologically active form. TH plays crucial role in regulating different aspects of animal physiology. The major role played by TH is regulation of metabolism, cellular growth, and development [3, 4]. However, recent advances in medical and molecular fields have helped to further dissect its other important role and mechanisms of action. TH has been shown to regulate nervous system differentiation as it influences neurogenesis, neuronal migration, neuronal and glial differentiation, myelination, and synaptogenesis [5–8]. Insufficiency in TH can lead to problems in cognition, visual attention, visual processing, motor skills, language, and memory skills [9]. TH is also implicated in neuropsychiatric disorders such as schizophrenia, bipolar disorder, anxiety, and depression [10, 11]. However, the molecular mechanisms of TH-mediated regulation of neuronal cells in these disorders are largely unknown. Some of the neurological diseases including Alzheimer’s disease (AD), Parkinson’s disease, and depression show a clear sex-specific incidence [12]. Moreover, thyroid-stimulating hormone (TSH) level has been associated with increased risk of dementia [13], and TSH level in plasma has become a routine screening test for diagnosis of patients with suspected dementia [14]. Low and high TSH has been associated with an increased risk of developing AD in women [15]. This suggests that elucidation of TH regulation and mechanisms of action in both male and female brains could further help to understand neuronal differentiation as well as neurological disease pathogenesis.

## TH production, transport, and mechanisms of action

THs are synthesized by the thyroid gland and circulated via blood, but tissue deiodinase enzymes play a critical role in regulating their levels inside the tissues [16]. There are three different types of iodothyronine deiodinase enzymes involved in TH regulation, namely DIO1, DIO2, and DIO3. DIO2 converts the pro-hormone, tetraiodothyronine or thyroxine (T4), into the biologically active form, triiodothyronine (T3), whereas deiodinase type 3 enzyme (DIO3) catalyzes the inactivation of T3 and T4. DIO1 can both activate and inactivate thyroid hormone and shows non-selectivity and high Km (requires a supraphysiological level of the substrate) for the conversion of T4 to T3 [17, 18].

Although T3 and T4 are lipophilic, they cannot cross the plasma membrane without the help of a transporter. There are different transporters including the monocarboxylate transporter (MCT) family (MCT8/SLC16A2 and MCT10/SLC16A10) and organic anion transporter polypeptide (OATP) family (SLCO1C1and OATP1C1) that are involved in TH transfer in and out of the cell [19]. In mice, the role of *Mct8* is considered to be more relevant than *Mct10* as *Mct8* knockout mice showed altered tissue homeostasis and serum T3 and T4 levels compared to Mct10 knockout mice [20]. *MCT8* gene inactivation in humans can lead to Allan-Herndon-Dudley syndrome, a condition where patients show severe neurological problems [21, 22]. Interestingly, *Mct8* gene knockout in mice does not show severe phenotype as in humans and this could be due to the availability of T4 through *Oatp1c1* transporter and its conversion to T3 at the cellular level [20].

TH action is mainly exerted by interaction with TH and thyroid hormone receptors (THRs) which are mainly of two types, *THRα* and *THRβ*. THR is a nuclear receptor that requires TH as a ligand to be activated. THR binds to thyroid hormone response element (TRE) on the gene promoter and generally forms hetero dimer with retinoid X receptor (RXR) [23]. The TRE sequence consists of two consensus half sites, AGGT/ACA, arranged either as direct repeat, palindrome, or inverted palindrome. The RXR binds to 5′ half site while THR binds 3′ half site [24].

**Fig. 1:**
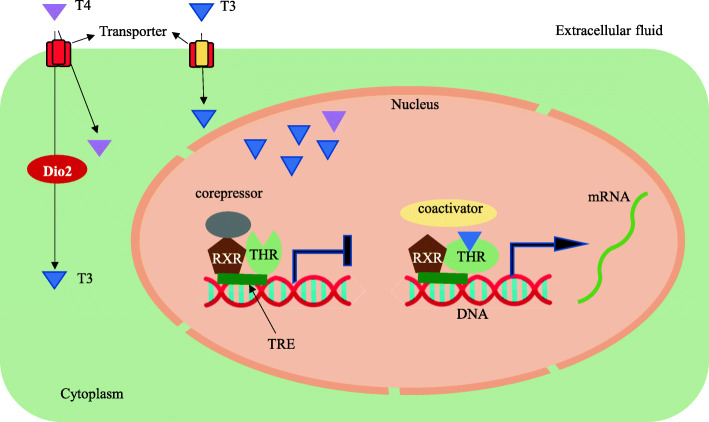
Mechanism of TH intracellular action. The introduction of TH into the cell is mediated by transporters. T4 can be converted into T3 by the enzyme, DIO2. DIO1 is also involved in the conversion of T4 (not shown in the figure). T3 and T4 bind to the THRs mainly associated with RXR, and this binding leads to the conformational change of THRs followed by dissociation of corepressors and recruitment of coactivators. This leads to the expression of genes that are located downstream of TREs

In the absence of TH, corepressors associate with THR to inhibit gene transcription. The binding of TH to the THR facilitates conformational change of THR, dissociation of corepressors from THR, and recruitment of coactivators, and this, in turn, drives gene transcription [4, 25, 26] (Fig. 1). The two half sites are generally separated by four nucleotides (DR4); however, other combinations are also reported. Among THs, T3 has approximately 10-fold higher binding affinity to THRs compared to T4 [25].

In addition to genomic effects of TH, non-genomic (transcription-independent/TRE independent) signaling has also been reported [27–31]. Compared to the genomic, the non-genomic action is rapid which takes place within seconds or minutes [28, 32]. Initially, it was noted that T3 can bind to rat erythrocytes membranes and mitochondrial fractions from rat liver [33, 34]. Later non-genomic effects of TH were reported for production of ATP, consumption of oxygen, activation of Na+/H+ exchanger, and increase of intracellular pH [35, 36].

The non-genomic action was suggested to be important for maintaining cell homeostasis by regulating ion concentration and cytoskeleton; however, the presence of crosstalk between genomic and non-genomic activities of TH is also proposed, which implies that the TH molecular mechanisms of action is diverse and complex [29]. The non-genomic action can initiate either at the cell membrane or in the cytoplasm, but the molecular mechanisms are not understood properly [31]. The cell surface receptor is generated from the internal translation initiation site of THRα which then gets palmitoylated and associates with caveolin-containing plasma membrane domains [27]. It is also shown that TH can mediate non-genomic activity via surface receptors αVβ3 integrin. TH action via αVβ3 leads to activation of *FGF2*, *HIF1α*, *COX2*, *THRA*, *THRB*, *ESR1*, *MMP9*, *NOS2*, *SREBP1*, and *CD74* genes while the expressions of *CASP3*, *BBC3*, *PMAIP*, and *APAF1* are downregulated [30]. The non-genomic activity is considered to be stronger for T4; however, it is not certain whether T3 or T4 acts on αVβ3 to regulate these genes [30]. It is not reported if the non-genomic action of TH can facilitate sex-specific signaling. However, based on the documented roles, it can be suggested that non-genomic action could be involved in differential signaling in males and females. For instance, TH crosstalk between genomic and non-genomic actions has been indicated for immune regulation [29], and since the immune system of males and females show sharp contrast [37–39], a sex-specific effect of TH on immune system via non-genomic action can be expected. Non-genomic action of TH is also considered to be important for brain development as T4 has been shown to alter actin polymerization and neural migration [40]. It is also suggested that activation of protein kinase Akt and endothelial nitric oxide synthase via T3 non-genomic action in rat brain could contribute to neuroprotective effects of TH [28]. Further investigation of TH non-genomic action in brain development will help to understand molecular mechanisms of TH in sex-specific regulation of neuronal signaling.

The production and secretion of THs are regulated by hypothalamus-pituitary-thyroid (HPT) axis. The hypothalamus (medial region of the paraventricular nucleus) synthesizes thyrotropin-releasing hormone (TRH) that enters the pituitary portal circulation. In the anterior pituitary, TRH stimulates the release of thyroid-stimulating hormone (TSH). TSH then travels to the thyroid gland where it stimulates the thyroid gland to secrete TH. The TH released in the circulation can regulate the level of TRH and TSH in the blood by negative feedback loop [41, 42].

## TH receptors (THRs) and distribution in the brain

**Fig. 2:**
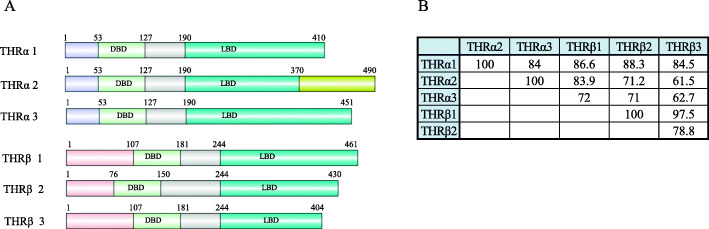
Thyroid hormone receptors. In humans, there are three isoforms of THRα and three isoforms of THRβ. The human protein sequence was obtained from NCBI, and the protein domain was prepared using the protein illustrator software DOG 1.0 [45] (**a**). The DNA binding domain (DBD) is highly conserved among the receptors; however, the ligand binding domain (LBD) shows differences in sequence and length (**b**)

TH mainly mediates its action by binding to the THRs in the cell cytoplasm. The THRs belong to the nuclear receptor superfamily, and there are two different types of THRs, *THRα* and *THRβ*. The protein sequence comparison using CLUSTLW showed that there is 62.3% similarity between THRα (451 aa) and THRβ (461 aa). Although they share structural and sequence similarities, mutation in one cannot fully compensate for the loss of another [31], and patients with mutations in either *THRα* or *THRβ* have strikingly different clinical phenotypes [43]. Patients and mutant mice for *THRβ* show large goiter and hearing impairment deregulation of HPT [43] axis while *Thrα* mutation in mice exhibits increased mortality, reduced fertility, and dwarfism [44]. In mammals, different TH receptor isoforms have been identified; for instance, in humans, 3 isoforms of THRα (THRα1, THRα2, and THRα3) and 3 isoforms of THRβ (THRβ1, THRβ2, and THRβ3) were found in NCBI database (Fig. 2). THRα expression is observed throughout the brain whereas THRβ is mainly expressed in the subcortical region of the brain [46]. TH regulation is critically regulated by the expression of THRα and THRβ.

**Fig. 3:**
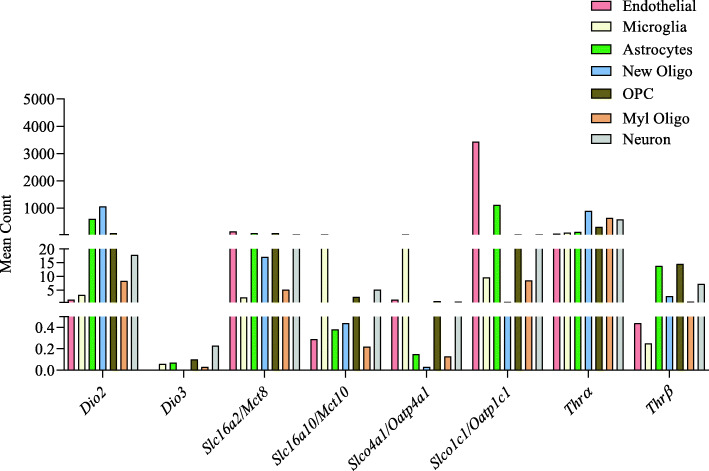
Expression of genes involved in different brain cells. RNA sequencing data was obtained from a previous study [47] and analyzed using Partek Flow software. The expression of genes involved in TH regulation was analyzed for mouse brain cells including endothelial, microglia, astrocytes, newly formed oligodendrocytes (New Oligo), oligodendrocyte precursor cells (OPC), myelinating oligodendrocyte (Myl Oligo), and neurons. *n* = 2 for all cell types

In mouse brain, *Thr*α and *Thr*β expressions were observed in different cells including endothelial cells, microglia, astrocytes, oligodendrocytes, and neurons. In these cells, the expression of *Thr*α was higher than that of *Thr*β with endothelial and microglia showing the lowest expression (Fig. 3).

## TH regulation and action in the brain

The active T3 and T4 produced in the thyroid gland enter the blood circulation which then gets distributed to different body parts. TH uptake in the brain is a slower process compared to other organs and is tightly regulated. Both T4 and T3 can cross blood-brain barrier (BBB) and enter the brain. TH transporter solute carrier family 16 member 2 (SLC16A2/MCT-8) and solute carrier organic anion transporter family member 1C1 (OATP1C1/SLCO1C1) are both present in the endothelial cells of BBB [48]. OATP1C1 is a T4 transporter; however, MCT8 can transport both T3 and T4 [19]. From the endothelial cells at the blood-brain barrier, T4 is transported to the astrocytes via membrane transporter OATP1c1, and in the astrocyte, T4 gets converted to T3 by the DIO2 enzyme. The expression of *Oatp1c1* was higher in the endothelial cells followed by astrocytes (Fig. 3). The expression of other transporters (*Slc16a2/Mct8* and *Slc16a10/Mct10*) (Fig. 3) could also provide important clues on brain cell regulation by TH. In the brain, the released T4 is taken up by the astrocytes and gets converted into an active form triiodothyronine, T3 [49]. It is indicated that the development of certain parts of the brain is dependent on the expression of deiodinases that convert T4 to more active T3 [2].

There is differential expression of iodothyronine deiodinases in different brain regions [19]. Dio1 is mainly active in mice cerebellum [50] and mostly absent in other brain regions, making Dio2 and Dio3 the major iodothyronine deiodinases in the brain. Deiodinases are membrane-bound proteins [51]. DIO2 is mainly located in the endoplasmic reticulum, and its catalytic domain is exposed to the ER lumen, whereas DIO1 and DIO3 are located in the plasma membrane having a catalytic domain exposed to the cytosolic side [52]. DIO2 is mainly expressed in astrocytes and DIO3 being mostly expressed in neurons. Sonic hedgehog (SHH) is a common regulator of both deiodinases. SHH induces DIO3 mRNA expression whereas it degrades DIO2 at the protein level via ubiquitination by WD repeat and SOCS box-containing box 1 (WSB1) [53, 54].

**Fig. 4:**
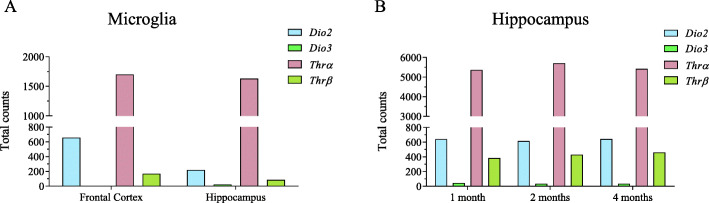
Expression of TH receptors and TH-synthesizing genes. The RNA seq data was obtained from NCBI submitted by a previous study [55]. Raw data were aligned and normalized using Partek Flow software. Expression analysis suggests that Dio2 expression is high in mouse microglia from cortex while Dio3 expression is high in the hippocampus. Thrα expression was found to higher than Thrβ expression. *n* = 3 males and *n* = 4 females (**a**). We further analyzed RNA seq data from another study [56] and observed that the expression of Dio2, Dio3, Thrα, and Thrβ in the mouse hippocampus is not strikingly different for the different developmental stages, *n*= 5 (**b**)

In our analysis, we observed that *DIO2* and *DIO3* genes are expressed in both male and female brains. Although the expression of these two genes was high in female microglia (*DIO2* 1.7 fold, *p* value 0.05, and *DIO3* 1.4 fold, *p* value 0.02), it was not significant after FDR adjustment. Comparison of counts showed that *DIO2* expression is higher than *DIO3* expression in both mice (Fig. 4) and human brains (*DIO2* mean count 350 and *DIO3* mean count 38). It is indicated that the source of T3 in microglia is astrocytes [11]; however, the presence of *DIO2* suggests that microglia can produce T3 locally. This data should be confirmed with DIO2 protein and T3 level analysis. In the developing rat brain, the expression of *THRα* is higher than that of *THRβ* [57]. Analysis of transcriptomic data of human fetal brain from a recent study [58] also showed that *THRα* (transcript mean count 5320) expression is higher than *THRβ* (transcript mean count 614) expression. Interestingly, it was shown that the negative outcome of hypothyroidism is not due to the lack of either *Thrα* or *Thrβ*, but due to decreased level of T3 in the circulation [59]. Using the knockout mouse model for *Thrα* or *Thrβ*, the authors analyzed gene expression in the cerebral cortex and the striatum and showed that individual knockout of either gene does not show marked differences and the two genes can largely compensate for each other’s loss [59]. The knockout mouse model for T3 transport, *monocarboxylate transporter 8* (*Mct8*) and *Dio2*, provided important information on localized T3 synthesis [60]. Inactivation of Mct8 showed limited effect on cerebral cortex gene expression postnatally. The authors suggested that this could be due to upregulation of Dio2 and local increase of T3 as the double knockout of *Dio2* showed similar effects as hypothyroidism [60].

Transcriptional repressor or activators can also regulate TH-dependent signaling. Nuclear receptor corepressor 1 (NCOR1) is identified as the key corepressor of TH-regulated genes in mice hepatic tissue [61]. In the same study, deletion of NCOR2 did not show significant changes in global TH signaling. However, the expression of NCOR1 during brain development is not well studied. T3-dependent transcription through TRE is abolished in mediator complex subunit 1 (MED1) null cells which suggest the possible role of MED1 as an activator for T3-dependent transcription [62]. Hence, MED1 shows the opposite activity of NCOR1 in TH-dependent gene expression.

T3 is critical for microglia development, and it can also induce microglial migration and phagocytosis. Microglia are immune cells of the brain that are involved in maintaining brain homeostasis and implicated in disease and injury [63]. Microglia are also involved in the regulation of neural functions and sexual behavior [64, 65]. The study by Guneykaya et al. show that the microglia in males are more frequent in specific brain areas and appear to have a higher potential to respond to stimuli [55]. It is also indicated that T3 can regulate morphological maturation of ameoboid microglial cells and limit their degeneration [66]. Decreased level of TH has been shown to reduce microglial processes in postnatal rats [67]. Given the importance of microglia in brain development, sexual behavior, and its regulation by TH, it can be speculated that brain sexual differentiation or sex-specific brain organization may be regulated by TH-mediated microglial functions.

Insufficient TH signaling could result in arborization of Purkinje cells, delay in neuronal migration, outgrowth of neuronal processes, myelination, and synaptogenesis [68, 69]. TH deficiency also leads to neuronal death and glial cell proliferation [70]. Low perinatal TH levels result in reduced dendritic complexity in Purkinje cells in the cerebellum [71]. This suggests an important role of TH for the cerebellar motor function, and dysregulation of TH in the perinatal phase can have long-term effects. TH in the early developmental phase also regulates GABAergic neuron morphology and connectivity via control of TrkB and mTOR signaling [72]. Synthesis of GABA from glutamate is regulated by glutamate decarboxylase 65 and 67 (GAD65 and GAD67). In vivo and in vitro data suggest TH regulates the expression of GAD enzymes in the brain [73] thereby regulating GABA production. TH deficiency in early rat developmental phase causes reduction in parvalbumin (PV)-positive neurons suggesting TH are also involved in early cortical circuit development [74].

TH in the brain regulates several pathways that contribute to structural aspects during development such as neurogenesis, cell migration, and myelination. TH is mainly involved in later events of neural development including neural migration or neuron-glia differentiation [19]. TH has been linked to adult neurogenesis [75], and it mainly occurs in two regions in the brain, namely the subventricular and subgranular zones, and is generally associated with cognitive deficits, psychiatric conditions, and depression [76]. TH administration stimulates neurogenesis in these two brain regions, whereas hypothyroidism inhibits neurogenesis [77, 78].

TH can have effects on cell migration in different brain regions like the cerebellum, hippocampus, and cerebral cortex [76]. TH is responsible for formation of different layer patterns; this migration is achieved by regulation of genes RELN and PTGDS by TH [79, 80]. Hypothyroidism causes poor myelination; on the other hand, hyperthyroidism increases myelination [81–83]. Hyper and hypothyroidism show different sex-specific phenotype [84]. Behavioral activity including locomotor activity, water intake, motor coordination, and muscle strength showed sex-specific alteration in thyroid dysfunction mice in this study [84]. In order to understand TH role in brain development, different mutant animals have been studied. For instance, congenital hypothyroid mice (*cog/cog* mouse) with mutation in thyroglobulin (Tg) gene show significantly low cerebrum and cerebellar weight [85]. Mutation in the same gene (Tg) in rat (*rdw* rat) shows altered dopamine level in the substantia nigra and striatum, impaired motor coordination, retarded cerebellar morphogenesis, retarded migration of granule cells, and poor dendritic aborization of Purkinje cells [86]. Interestingly, the analyzed parameters in this study showed sex-specific differences. The motor coordination and balance measurement using the rotarod test on this rat model showed that *rdw* female and male rats, respectively, showed a 15% and 5% decrease in activity compared to wild type female and male rats. Among other parameters, rearing behavior in *rdw* rats were significantly decreased compared to female rats. The dopamine level in the substantia nigra was increased to around 1.6 fold in females while it increased 1.9 fold in males. On the other hand, dopamine in the striatum decreased by 1.5 fold in females while it decreased to 1.2 fold in males [86]. Mutation in *Dual oxidase 2* (DUOX2) gene in humans leads to congenital hypothyroidism [87]. *DUOX2* is involved in generation of hydrogen peroxide, which will then be utilized by thyroid peroxidase for iodine incorporation into thyroglobulin [88]. Mouse *Duox2* mutant shows severe hypothyroidism and hearing impairment [88]. Models with *Thrα* or *Thrβ* mutation have also been studied to understand brain function; however, the phenotype of *Thr* mutant is different from hypothyroid models. Since the expression of *Thrα* is high in the brain compared to *Thrβ* (also observed in our study; Fig 3), severe phenotype is expected with *Thrα* mutation [89]. In addition, deletion of *Thrα* or *Thrβ* leads to different phenotypes as it has been shown that deletion of *Thrα1* in mice reduces female sexual behavior while deletion of *Thrβ* increases it when stimulated with estrogen [90]. However, the impact of male sexual behavior was not evaluated in this study. Another study showed that deletion of *Thrα1* in male mice altered exploratory behavior, decreased rearing behavior, and increased freezing behavior [91]. This behavior change was linked to altered hippocampal signaling [91].

## Sex-specific effects of TH in the brain

Sex differences in the brain regulate not only reproductive functions but also cognitive abilities and susceptibility to neurological diseases. In mammals, gonadal steroid hormone surge during the fetal stage organizes the brain, and later during the adult stage, the second surge of gonadal hormone leads to behavioral activation. The classical model of brain sex differentiation suggests that the gonadal steroid hormones (androgens and estrogens) are the main drivers in establishing male and female neural networks [92, 93]. Although the role of steroid hormones is critical in organizing the brain in a male- and female-specific manner, the involvement of other key players including TH cannot be overruled.

The thyroid-related medical problems including hypothyroidism and hyperthyroidism are more common in females than males [94]. Transcriptomics analysis also revealed that aging-related changes in thyroid tissue are more common in females [95].

TH could have sex- and age-dependent effects as it has been shown that exposure of T4 to male mice results in activation of glial cells while that to female mice leads to deactivation [11]. Comparison of glial activation following exposure to T4 in young mice brain showed sex-specific effects. In males, T4 exposure activated glial cells while in females it deactivated them [11].

Critical information on sex-specific role of thyroid hormone came from songbird, zebra finch (*Taeniopygia guttata*). In this bird, the levels of T3 and T4 in the brain and plasma have been shown to be sex specific with male and female showing different peak periods [96].

The level of *Dio2* mRNA was shown to increase at 21 days post-hatching (dph), and the level of T3 also started to increase after 21 dph in male brain [96]. In addition, Raymaekers et al. showed that the level of *Dio2* is higher in the male song control nuclei [97]. The increase in *Dio2* and T3 corresponds to the timing when zebra finch males learn to sing [96]. This suggests that TH is crucial for male typical brain development in zebra finches. Taken together, it can be suggested that TH could have sex-specific role in brain development.

In P0 (perinatal day 0) neonatal rats, Dio3 expression was transiently noted in regions involved in sexual differentiation in the brain. This expression was not observed in P10 rats, this suggests the role of Dio3 in early sexual differentiation in rodents [98]. There was sex-specific difference in Dio1 levels in mice. Expression of Dio1 in both pituitary and thyroid glands were higher in adult males compared to females [99]. However, there was no significant difference in TH levels between sexes in the same study.

SHH has been identified as a common regulator of both DIO2 and DIO3 [53, 54]. It induces DIO3 whereas degrades DIO2 and thereby plays an important role in maintaining balance of TH in intracellular context. SHH receptor patched1 (PTCH1) haplosufficiency shows sex-specific effect and female-specific reduction in hippocampus size and isocortical layer thickness [100]. It would be interesting to study whether SHH signaling can have sexually dimorphic effect in brain TH regulation.

Gould et al. noted in adult rats that females possessed more primary dendrites, whereas males showed more apical excrescences in CA3 pyramidal cells. TH treatment resulted in increased primary dendrites as well as apical excrescences in both sexes [101]. Sex difference was noted between serum TSH levels and depressive symptoms in cohort with normal serum T4 levels. Higher TSH level was correlated with higher prevalence of depressive symptoms in men whereas the opposite was noted for women [102].

To investigate whether thyroid signaling is differentially regulated in male and female brains, we analyzed different transcriptomics data that were available in the NCBI database deposited by previous studies [55, 56]. Analysis of male and female microglia from 1-, 2-, and 4-month-old mice did not show any strong differences. In all the three stages, the expression pattern showed a similar trend with higher expression of *Dio2* compared to *Dio3* and higher expression of *Thrα* than *Thrβ* (Fig. 4).

**Fig. 5:**
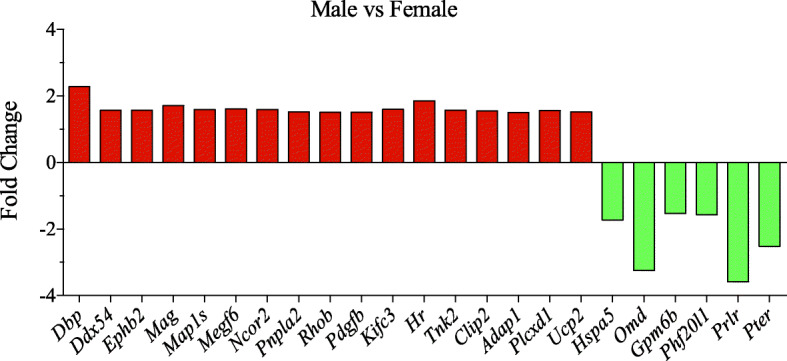
THR response genes are differentially regulated in male and female microglia. RNA seq data was obtained from a previous study [55] and analyzed using Partek Flow software. The microglia from hippocampus showed sex-biased differences in the expression of TH-regulated genes. Fold change ± 1.5, *p* value 0.05, and FDR 0.05. *n* = 3 males and *n* = 4 females

Analysis of transcriptomics data of microglia from the frontal cortex and the hippocampus of adult mice was also analyzed. In the frontal cortex, only 8 genes showed significant difference, and out of these, one gene, *Dbp*, is a T3-regulated gene [103]. Interestingly, the microglia from the hippocampus showed 1386 differentially regulated genes between males and females, and from this list, we extracted the T3-regulated genes (Fig. 5). Out of 23 regulated genes, 17 genes were high in males and 6 genes were high in females. The genes including THR, TH synthesis, and transport were not differentially regulated. Critical links are missing to understand the mechanisms of how TH can differentially regulate gene expression in male vs female brain.

## Thyroid hormone in neurodegenerative and psychiatric diseases

Several neurological and psychiatric conditions are associated with TH dysregulation. Hypothyroidism during pregnancy increases risk of autism, cognitive impairment, and attention deficits [46]. On the other hand, hyperthyroidism is known to cause anxiety, hyperflexia, and irritability. Both hyper and hypothyroidism are associated with mood-related conditions, personality disorders, and dementia [76]. Hypothyroidism has been shown to induce interleukin 1 (IL-1)-mediated autophagy and neuronal apoptosis in postnatal rats that accounts for cognitive impairment [104].

Females are more frequently affected by AD than males [105]; the same is observed in thyroid dysfunction diseases [94]. It is intriguing whether there is an underlying correlation between TH and AD onset/pathogenesis. Intebi et al. studied some of the plasma markers in an AD cohort; however, they could not identify any change in circulating T3 and TSH levels between male and female AD patients [106]. However, in another study, female sex and thyroid dysfunction were correlated with AD endophenotype in the middle-aged population [107]. Further mechanistic understanding is needed to have a clear view on this aspect.

Low serum T4 and upregulated serum TSH levels showed correlation with brain amyloid beta levels and AD-specific brain alterations [108]. T3 administration in diabetic mice decreased glycemia, improved insulin sensitivity, and reduced GSK3B activation as well as tau protein load in hippocampus [109]. This is considered beneficial since hyperphosphorylated Tau (MAPT) accumulation and GSK3B activation are hallmarks of AD [110]. Apolipoprotein E (APOE) more specifically isoform APOE4 is associated with AD [111, 112]. A study in older Down syndrome (DS) patients having AD suggests that APOE2 might protect against hypothyroidism; however, APOE4 predispose towards the same [113]. This effect is only observed in females, and no such correlation was noted for males in the same study. It is concluded that APOE4 pathogenesis in AD patients is partially affected by thyroid function [113].

Cerebrospinal fluid (CSF) T3 levels were found to be higher in hippocampal sclerosis (HS) but at a normal level in AD [114]. HS-associated SNP rs73069071 was associated with mRNA expression levels of astrocyte TH transporter SLCO1C1 [114]. Mutations in TH transporters like MCT8 (SLC16A2), and OATP1C1 (SLCO1C1) cause juvenile neurodegeneration and brain developmental disease, Allan-Herndon-Dudley syndrome. *oatp1c1* (*slco1c1*) knockout zebrafish also showed a similar phenotype [115, 116]. The function of TH in the context of myelination has been implicated in neurological disorders including multiple sclerosis (MS) to the extent that TH benefits MS by augmenting myelination [117, 118]. TH is often associated with antioxidant activities, and dysfunction of TH could increase reactive oxygen species (ROS) and, hence, oxidative stress which increases neurodegenerative mechanism in the brain [119]. T3 treatment showed neuroprotection in traumatic brain injury murine model [120]. This suggests that decreased TH level could predispose individual to ROS-mediated brain damage, and this, in turn, could aggravate the neurodegenerative outcome.

*DIO1* polymorphism is associated with serum TH level and temporal lobe atrophy in the elderly population [121]. Thr92Ala-DIO2 has been associated with increased risk for AD in various cohorts [122].

Although there is no direct correlation between Parkinson’s disease (PD) and TH, there are reports explaining the commonalities between Parkinsonism and thyroid dysfunction. In particular PD patients suffering from hypothyroidism, hormone therapy proved to be helpful in reducing Parkinson’s bradykinesia and hypomimia [123]. On the other hand hyperthyroidism increases tremor in PD cases, which can be managed by anti-thyroid treatment [124].

Crystalline mu (CRYM) is a regulator of T3 transportation [125]. It has been reported that CRYM expression in the striatum is reduced in Huntington’s disease (HD) mouse model and overexpression of CRYM reduced mutant Htt-mediated neurotoxicity [126]. This could be an important mechanism linking decreased TSH and T3 levels observed in HD patients [127].

Many studies have associated thyroid status with cognition, mood, and behavior. Thyroid dysfunction can lead to psychiatric changes without other symptoms of the disorder to the extent that hypothyroidism can be falsely presented as psychosis in older women [128]. Thyroid dysfunction is also noted in patients with schizophrenia spectrum disorders, bipolar disorder, and major depressive disorder [129]. Higher T3 and T4 and lower TSH levels were observed in schizophrenic patients [130]. The T3 levels in schizophrenics correlated significantly with plasma malondialdehyde and total plasma peroxides (TPP), which suggest higher TPP could contribute to better thyroid homeostasis in schizophrenia through regulation of free radicals and oxidative stress [130]. A strong correlation has been noted between anti-psychotic drug lithium and higher TSH and T4 and lower T3 levels in bipolar disorder patients [131]. The increased volume of thyroid gland following lithium treatment was also noted in the same study. Hypothyroidism is a common effect of long-term lithium treatment [132]. Thyroid dysfunction is more common in females than males, and this contributes to increased difficulties in diagnosis and treatment of mood disorders like bipolar disease [133]. Hypothyroidism is also noted in women with postpartum depression [134]. Presence of anti-thyroid auto-antibodies correlated with higher occurrence of panic disorder and major depressive disorder in a cohort of celiac disease [135]. Interestingly, higher serum TSH levels correlate with lower depressive symptoms in individuals with normal serum TH levels [102].

Thyroid-related diseases show sex-specific and age-dependent incidences with females showing 5–20 times higher susceptibility than males [136]. The underlying molecular mechanism is not clear but the difference in sex steroid milieu could be a critical determining factor. During menopause and andropause, the level of estrogen in females and testosterone in males drops down [137]. In males, low serum testosterone was associated with depression [138], low memory and cognitive skills [139], and risk of AD [140]. On the other, menopause in females decreases cognition and increases the chances for AD [141]. However, there are conflicting data whether hormone replacement therapy in women can prevent neurological diseases [141, 142]. This suggests that the neurological disease outcome in elderly people is multifactorial where TH could also play a crucial role. In aged men and women, the thyroid function decreases [143] and consequently it alters brain function [144] and increases AD risk in women [15]. In menopausal women with altered thyroid function, neurological problems including depression and anxiety are common [145]. Since the prevalence of thyroid-related and neurological diseases increase and steroid hormone level decreases with age, a positive correlation between sex steroid, thyroid dysfunction, and neurological diseases could be expected.

**Fig. 6:**
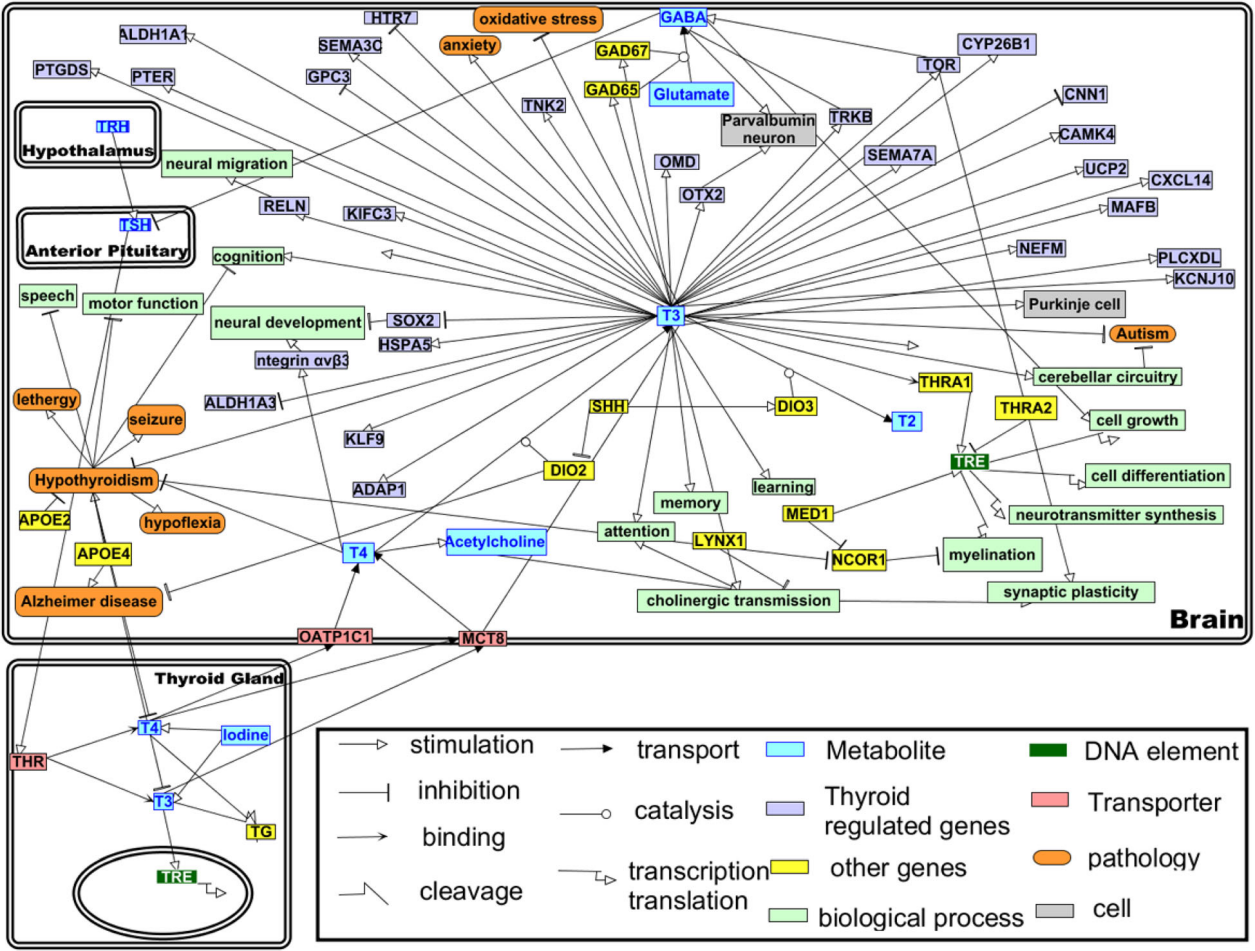
Thyroid hormone-related molecular mechanism in the brain: TH-related molecular causal relationships were extracted from the articles listed in the reference section of this manuscript, and the manually curated network was built on systems biology graphical notation (SBGN) [146] using Pathvisio 3.3.0 network editor [147]. Only major causal (cause-effect) relationships were used for the network; correlative relationships were not included in this model

Overall, there are many reports and indications that TH status is critical in several neurodegenerative and psychiatric diseases. However, the molecular mechanisms of the pathogenesis are not well elucidated. In Fig. 6, we have tried to assemble the known biomolecules and pathways associated with TH regulation and signaling. Mechanistic studies are required to have a better understanding of the involvement of TH in neurodegenerative and psychiatric diseases.

## Conclusion and future perspectives

TH regulates critical biological processes including brain differentiation. TH has been shown to regulate brain differentiation, and any alteration in level could lead to various nervous system-related problems. The common neurological problems associated with TH are cognition, visual attention, visual processing, motor skills, language, and memory skills. TH shows sex-specific effects in brain cell differentiation which could lead to differential organization of neural circuits. TH-related problems are also on the rise with females showing higher incidence. Our study suggests that there is clear sex-specific effects and regulation of TH in male and female brains. The sex-specific role of TH has started to emerge; however, critical links are missing to fully understand the molecular mechanisms. Understanding of TH sex-specific effects could further help to advance the diagnostic as well as the therapeutic field.

## Data Availability

Not applicable

## Abbreviations

AD: Alzheimer’s disease
APOE: Apolipoprotein E
BBB: Blood-brain barrier
CRYM: Crystalline mu
CSF: Cerebrospinal fluid
DIO: Iodothyronine deiodinase
DS: Down’s syndrome
HPT: Hypothalamus-pituitary-thyroid axis
HD: Huntington’s disease
MCT8: Solute carrier family 16 member 2 (SLC16A2)
OATP1C1: Solute carrier organic anion transporter family member 1C1 (SLCO1C1)
PD: Parkinson’s disease
ROS: Reactive oxygen species
RXR: Retinoid X receptor
TH: Thyroid hormone
THR: Thyroid hormone receptor
TRH: Thyrotropin-releasing hormone
TSH: Thyroid-stimulating hormone
T4: Thyroxine
T3: Triiodothyronine
TPP: Total plasma peroxides
SHH: Sonic hedgehog
